# Supplementary material for: Multiomics interrogation into HBV (Hepatitis B virus)-host interaction reveals novel coding potential in human genome, and identifies canonical and non-canonical proteins as host restriction factors against HBV
Source: Cell Discov. 2021 Nov 2;7:105. doi: 10.1038/s41421-021-00337-3 (PMC8560872; doi:10.1038/s41421-021-00337-3)
Supplement: Supplementary file 12 — Supplementary Figures [file 41421_2021_337_MOESM12_ESM.pdf]

Supplementary figures

Figure S1

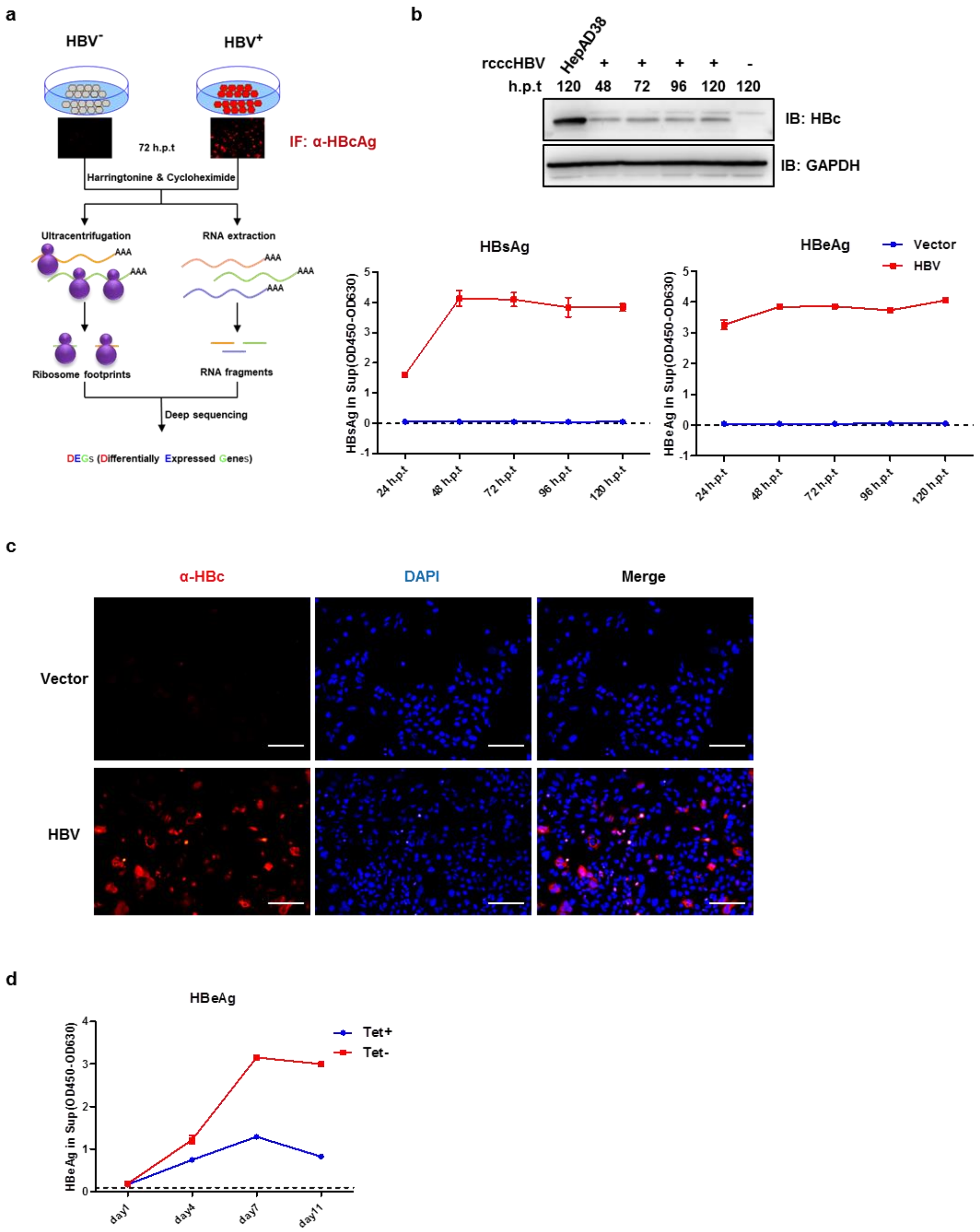

Figure S1: Replication kinetics of recombinant cccDNA system of hepatitis B virus. Huh7.5.1 cells were transfected with HBV recombinant cccDNA system (rcccHBV) or vector plus pCMV-Cre. (**a**) Experimental approaches for ribosome profiling of host genes upon transfection with the Cre-based rcccDNA system of HBV. (**b**) Cells were harvested at sequential time points as indicated, and lysates of HepAD38 cells which have been cultured for 120 hours after tetracycline withdraw

was used as a positive control. h.p.t: hour post transfection. Culture medium in vector or HBV group was harvested and supernatants collected at sequential time points as indicated were subjected to ELISA (n=2). (**c**) Cells were subjected to immunofluorescence assay at 72 h.p.t. using anti-HBcAg (Dako, B0586, red) and nuclei stained by DAPI (blue). Scale bars, 100 μm. (**d**) The expression of HBeAg secreted from HepAD38 cells chromosomally integrated with the Tet-inducible HBV expression system at indicated time points after tetracycline withdraw was accessed by ELISA (n=2).

Figure S2

a

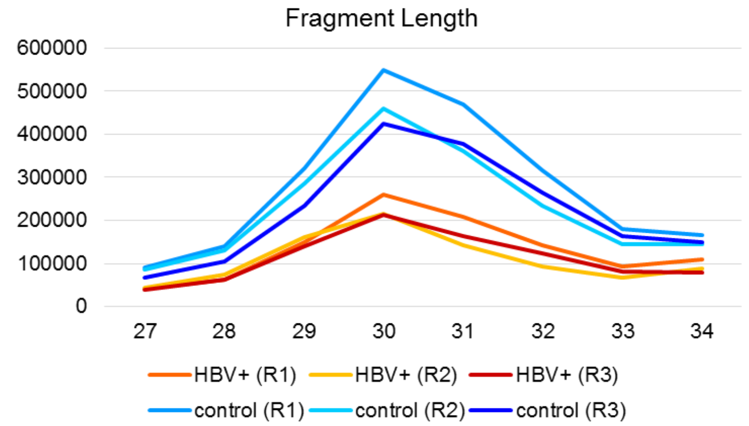

b

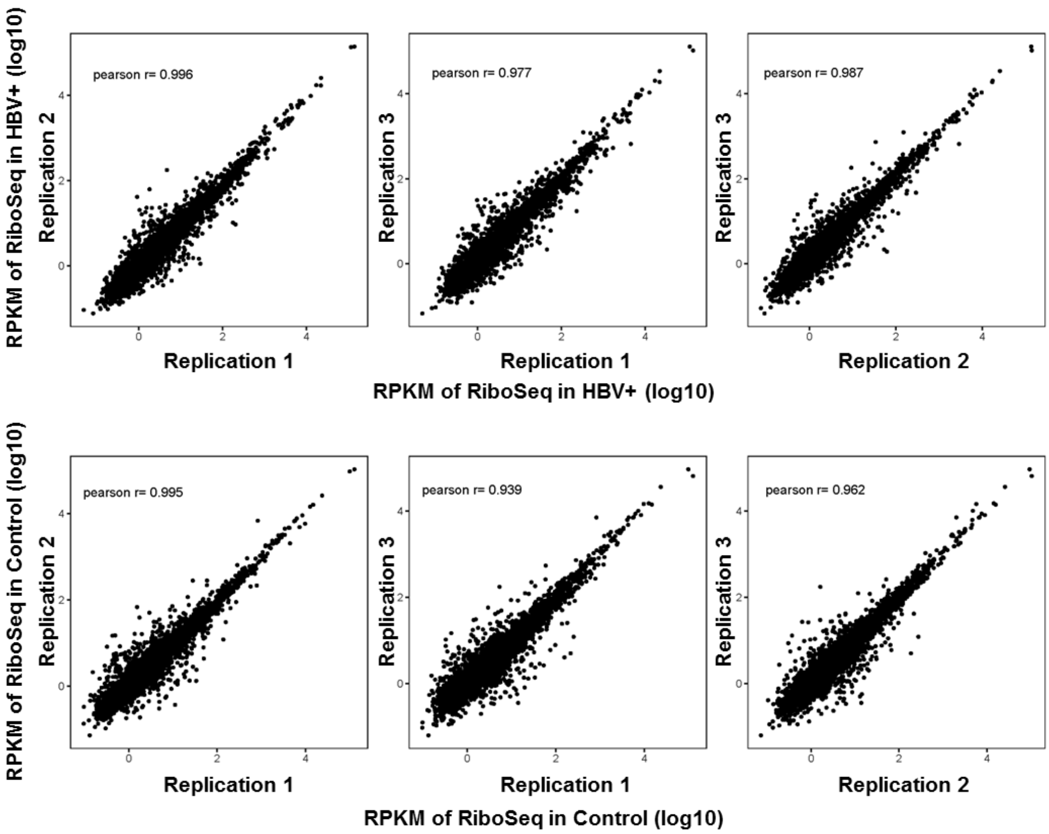

Figure S2: The length distribution of ribosome footprints and reproducibility of ribosome profiling experiments. (**a**) Control R1-3, replicate 1-3 in control group; HBV+ R1-3, HBV group replicates 1-3. X axis, the length distribution of ribosome footprints length in nucleotide (nt); Y axis, the reads numbers. (**b**) Plots show the correlations of RiboSeq RPKMs between three biological replicates in either HBV or non-HBV groups. Only mRNAs matched with > 5 reads were counted.

Figure S3

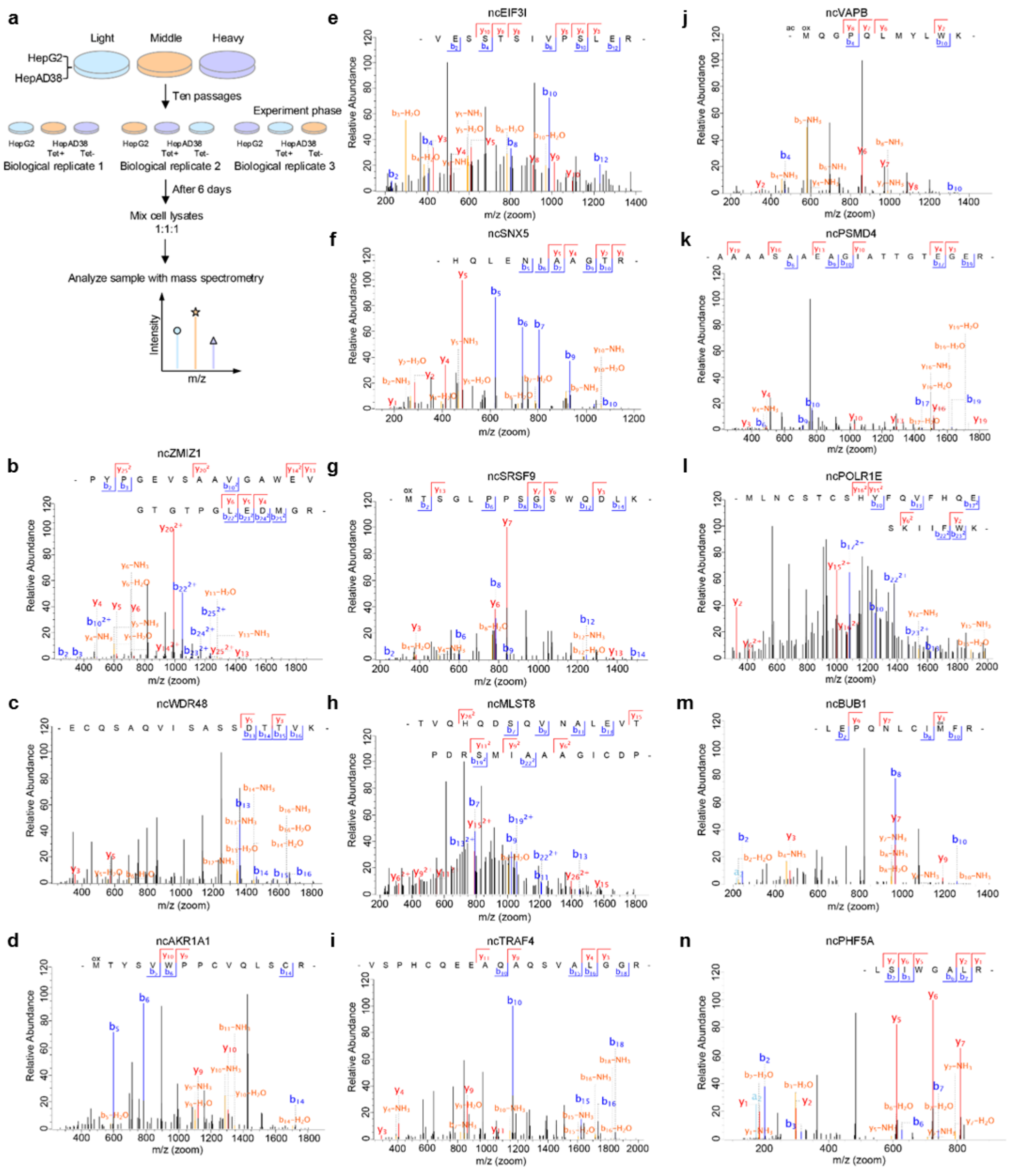

Figure S3: SILAC identified 13 peptides produced from non-canonical ORFs. (a) Three biological replicates were produced by labeling cells with different combination of light, middle and heavy stable isotopes, after experimental phase, cell lysates of each replicates were mixed by 1:1:1 and subjected to mass spectrometry (See online methods for more details). (b-n) MS spectra of identified peptide with sequence uniquely matched to the translated products of the non-canonical ORFs discovered in our ribosome profiling assays. These non-canonical ORFs were never annotated before and could have sequences and functions totally unrelated to any known proteins. They were nevertheless named after the closest annotated ORF in human genome. Matched y-ions and b-ions were shown in red and blue, respectively; the modified ions are shown in orange.

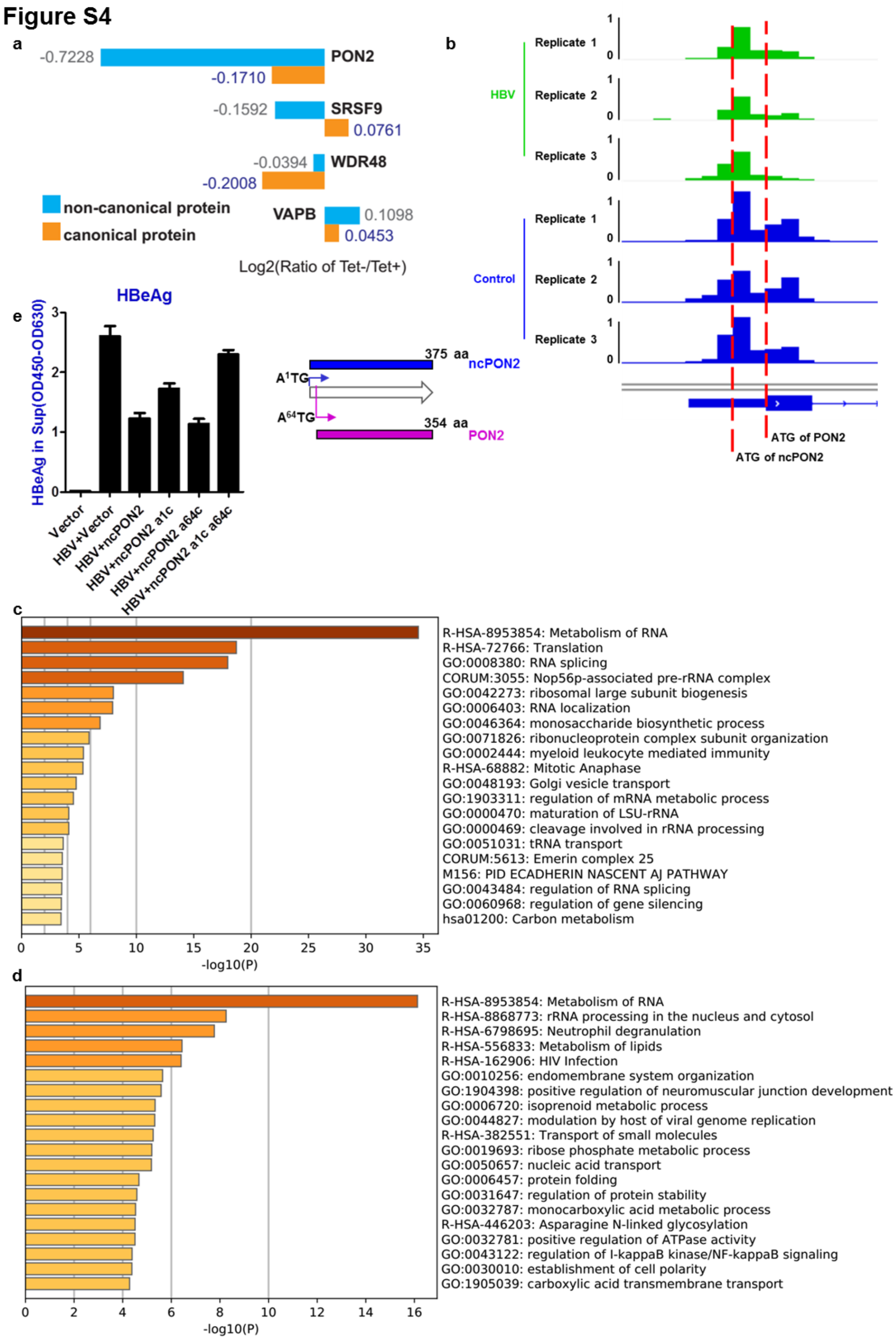

Figure S4: Analysis on ncPON2 and ncGRWD1.(**a**) The SILAC ratio of representative novel peptides and the corresponding canonical proteins in HBV+/HBV-, also see **Supplementary file S3 and S10**. (**b**)The IGV screen shot of RiboSeq pattern around the N-terminal region of *PON2* in all three biological replicates in either HBV or non-HBV groups. (**c** and **d**) To analyze the two HBV suppressive ncORFs, co-immunoprecipitation coupled with mass spectrometry

analysis was performed with ncGRWD1-FLAG and ncPON2-FLAG, with pCDNA3.0-FLAG as a negative control. And GO and pathway enrichment analysis was performed using a web server Metascape (http://metascape.org/) with the identified interactors of ncGRWD1 (**c**) or ncPON2 (**d**) (also see **Supplementary file S4**). (**e**) ***ncPON2 a1c*** denotated the construct in which the ATG start codon of ncPON2 was mutated to CTG, thus only supporting the expression of PON2; ***ncPON2 a64c*** , the construct that had the ATG start codon of canonical PON2 mutated to CTG, thus only expressing ncPON2; ***ncPON2 a1c a64c*** did not encode either PON2 or ncPON2. The data shows ***ncPON2 a1c*** (which only express canonical PON2) could inhibit HBeAg expression during HBV replication, though to a lesser extent compared to ***ncPON2*** and ***ncPON2 a64c*** (which only express ncPON2).

Figure S5

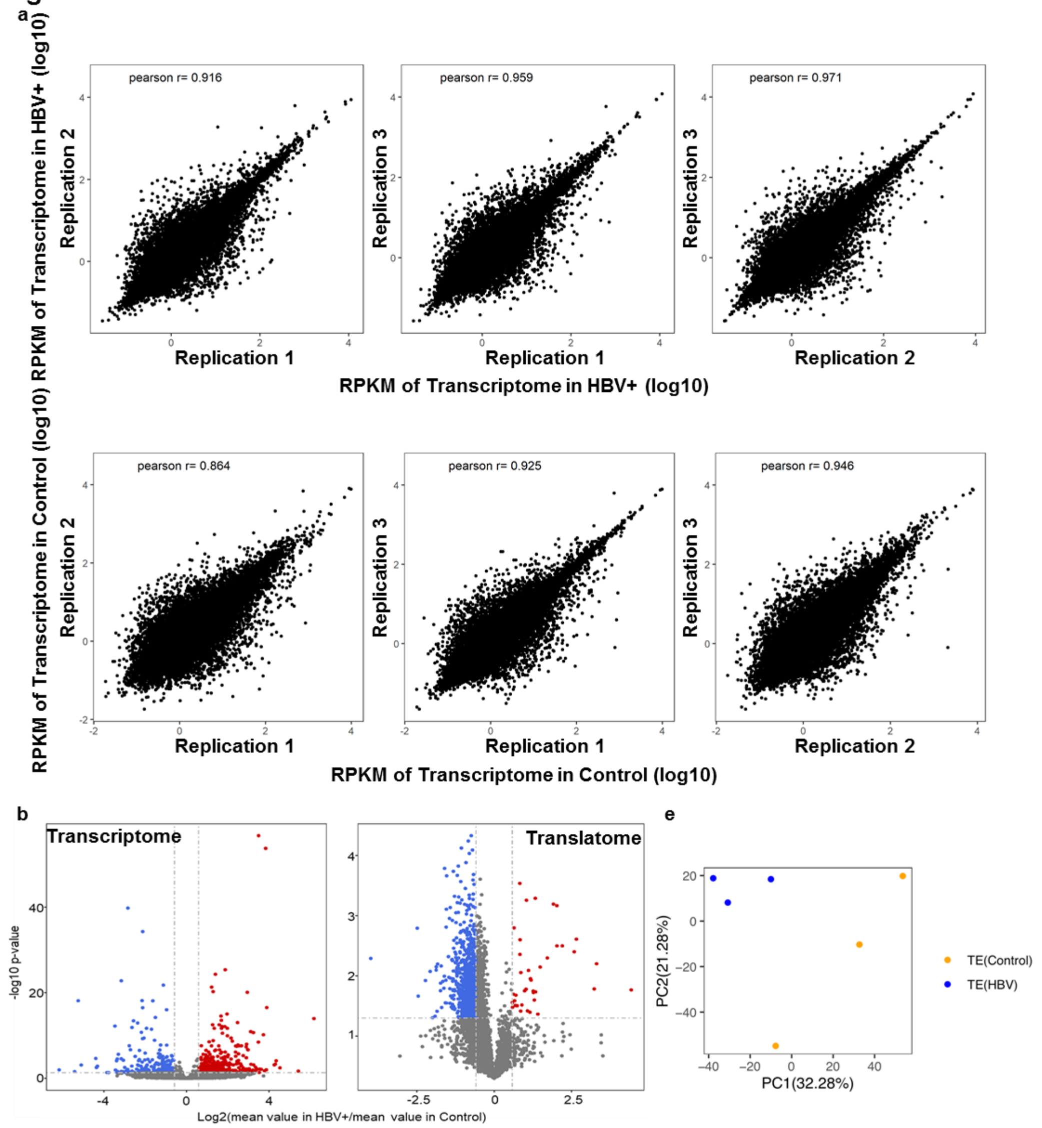

c KEGG enriched pathways of DEGs from transcriptome (pvalue<=0.01)

Term	pvalue	p.adjust	Count
Ribosome	1.45E-08	3.96E-06	21
Protein processing in endoplasmic reticulum	0.0001	0.0099	16
Spliceosome	0.0003	0.0313	13
Ferroptosis	0.0016	0.1071	6
Bacterial invasion of epithelial cells	0.0024	0.1316	8
Non-alcoholic fatty liver disease (NAFLD)	0.0029	0.1328	12
Cysteine and methionine metabolism	0.0036	0.1420	6
Central carbon metabolism in cancer	0.0045	0.1555	7
Fc gamma R-mediated phagocytosis	0.0085	0.2120	8
Biosynthesis of amino acids	0.0099	0.2259	7

d KEGG enriched pathways of DEGs from translatome (pvalue<=0.01)

Term	pvalue	p.adjust	Count
Necroptosis	0.0013	0.2655	17
TGF-beta signaling pathway	0.0029	0.2655	11
Cellular senescence	0.0030	0.2655	16
Hepatocellular carcinoma	0.0049	0.2655	16
Adherens junction	0.0058	0.2655	9
N-Glycan biosynthesis	0.0070	0.2655	7
Hippo signaling pathway - multiple species	0.0099	0.2655	5

Figure S5: Reproducibility assay, volcano plot and KEGG pathway analysis of RNA-seq and ribosome profiling experiments. (**a**) Plots show the correlations of RNA-seq RPKMs between three biological replicates in either HBV or non-HBV groups. Only mRNA matched with > 64 reads were counted. (**b**) Differentially expressed genes (DEGs) in transcriptome and translatome were depicted as volcano plot, with *p*-value and fold change both shown; up-regulated and down-regulated genes were depicted as red and blue, respectively. (**c**, **d**) KEGG pathway enrichment analysis of genes differentially transcribed (**c**) or translated (**d**) in HBV-loaded cells

versus control cells. (e) Principal-component analysis of global TE in HBV and control group, three biological replicates each.

Figure S6

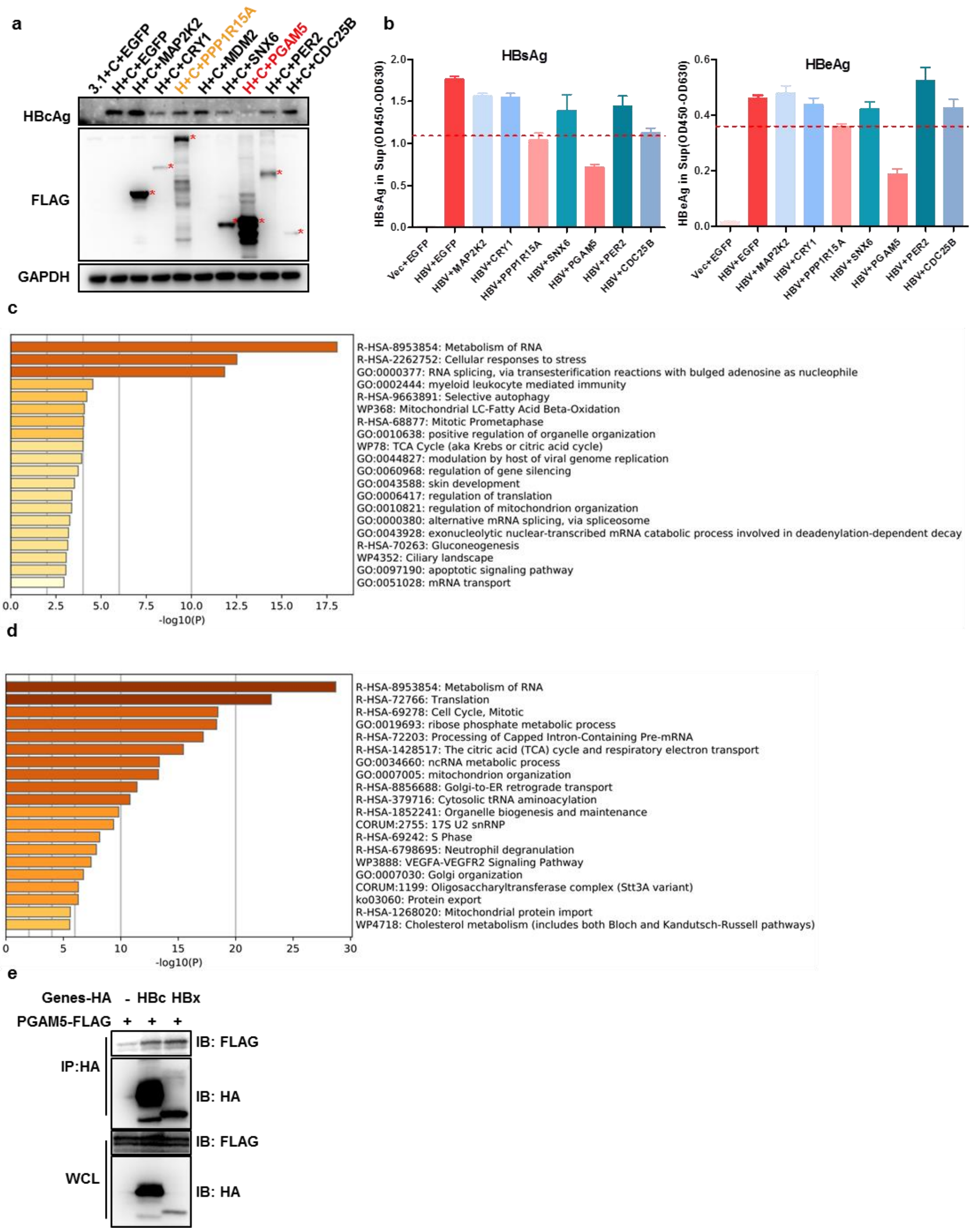

Figure S6: Screen and analysis on transcriptional and translational DEGs. (**a-b**) We tested the plasmids we have of the 35 transcriptional and translational DEGs as well as MDM2 in recombinant cccDNA system. Please note that we failed in detecting MDM2. (**c** and **d**) To further analysis the mechanism of the suppressive effect of PPP1R15A and PGAM5 on HBV, co-immunoprecipitation coupled with mass spectrometry analysis was performed with empty vector as a negative control to identify deemed interactions between host proteins and PPP1R15A or PGAM5, and

GO and pathway enrichment analysis was performed using a web server Metascape (http://metascape.org/) with the identified interactors of PPP1R15A (**c**) or PGAM5 (**d**), also see **Supplementary file S9**. (**e**) We performed co-immunoprecipitation between PGAM5 and two proteins which were essential for virus replication, HBc and HBx.

Figure S7

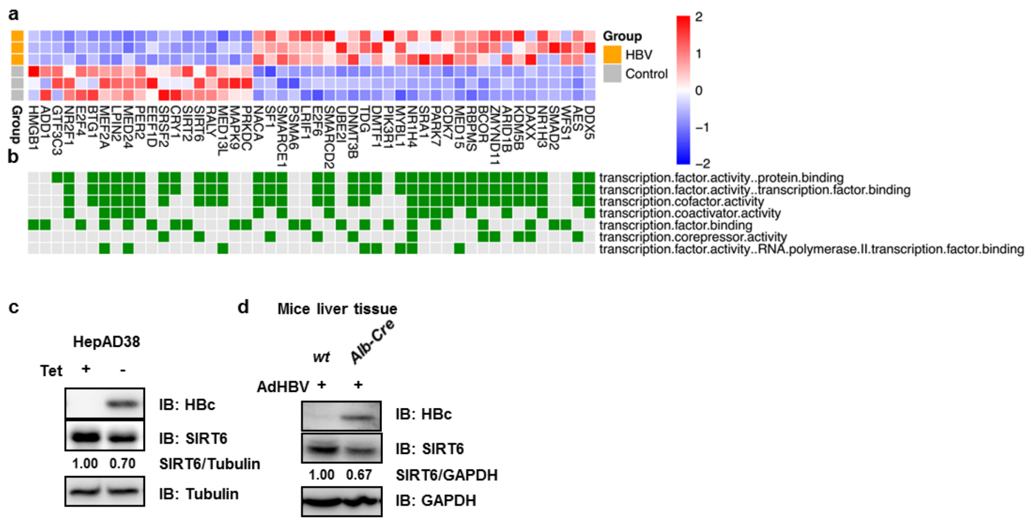

Figure S7: HBV down-regulates SIRT6 in HepAD38 cells and mouse model. (**a**) Heatmap of DEGs from RNA-seq in transcription molecular function. (**b**) The heatmap of DEGs participate in the indicated GO term. (**c**) HepAD38 cells with or without removal of tetracycline for 5 days were harvested, and lysates were subjected to IB analyses using indicated antibodies. (**d**) 14 days after recombinant adenovirus harboring HBV prcccDNA was injected into wild type or *Alb*-*Cre* transgenic mice, total proteins from the mouse liver tissues in each group were extracted and subjected to IB analyses using indicated antibodies.

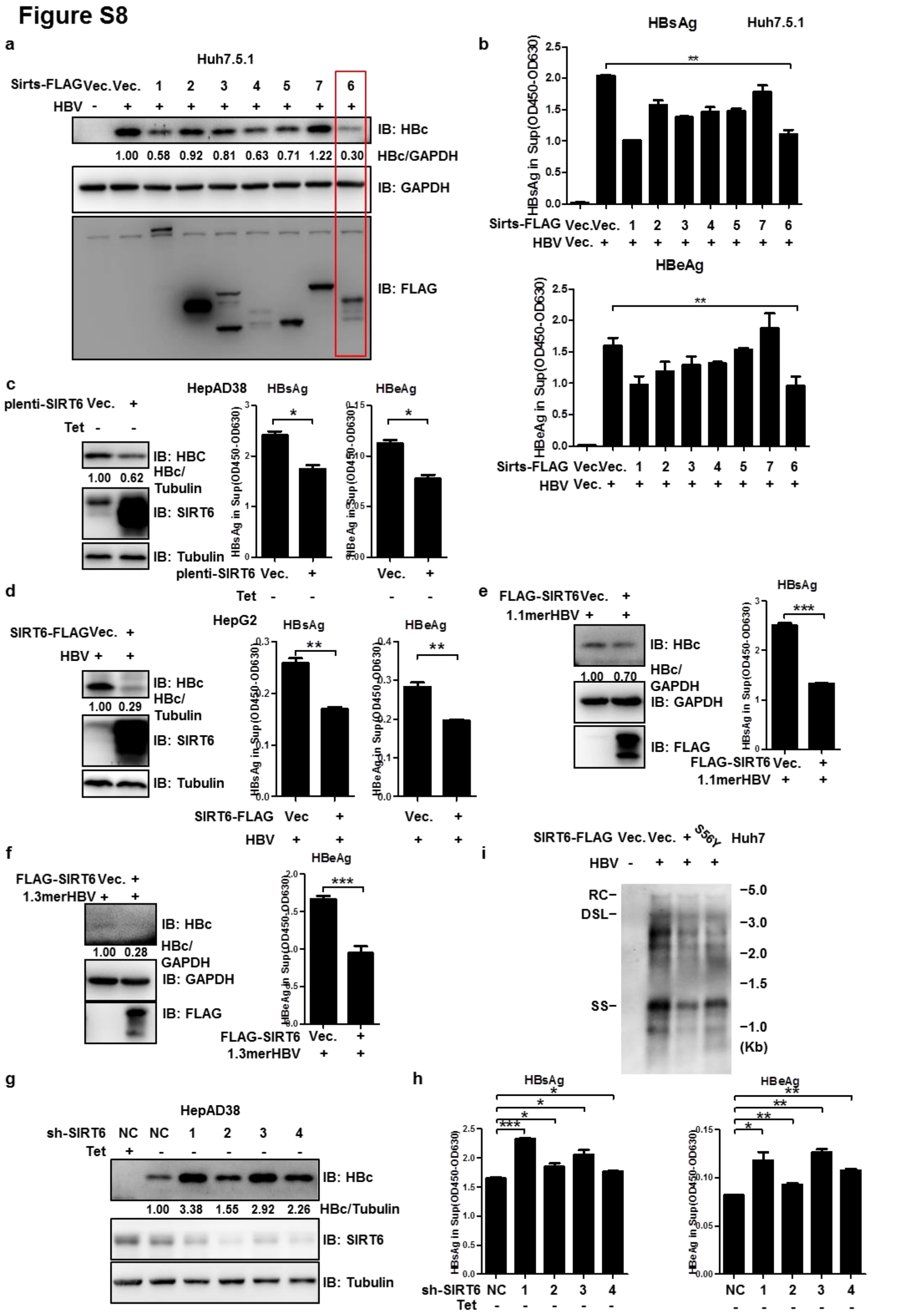

Figure S8: HBV down-regulates SIRT6 reciprocally in multiple HBV replication systems. (**a** and **b**)The cDNA of sirtuins-family were each co-transfected with HBV system into Huh7.5.1 cells and the protein levels of HBc, GAPDH and SIRTUINS were detected using indicated antibodies (a). The HBsAg and HBeAg level in supernatants were measured by ELISA. (b). (**c**) HepAD38 cells chromosomally

integrated with the Tet-controlled HBV expression system were infected with lenti-virus vector expressing SIRT6, Cells were harvested and lysates subjected to IB using the indicated antibodies, while the supernatants from the cell culture were collected and subjected to ELISA using anti-HBsAg or anti-HBeAg. (**d**) HepG2 cells were co-transfected with HBV cccDNA system (H+C, prCCCDNA and pCMV-Cre) and pCDNA3.0-Sirt6-FLAG or pCDNA3.0 using Polyetherimide . Cells were harvested 72 h.p.t., with lysates collected and subjected to IB analyses using the indicated antibodies. The HBsAg and HBeAg in supernatants were determined by ELISA. (**e** and **f**) Experiments similar to those in d were performed with Huh7.5.1 cells transfected with 1.1mer- (e) or 1.3mer- (f) HBV linear genomes, For ELISA, n=3. (**g**) HepAD38 cells were transduced with lenti-virus vector (NC) or those encoding shRNAs that targeted endogenous SIRT6 (shSIRT6 1,2,3,4), After tetracyclin withdrawn for 4 days. HBcAg was measured using western blot, and (**h**) the level of HBsAg and HBeAg in supernatants was determined by ELISA. (**i**) The effect of SIRT6 on HBV genome replication was measured via southern blotting in Huh7 cells. RC, relaxed circular DNA; DSL, double strand linear DNA; SS, single strand DNA. *, $p<0.05$, **, $p<0.01$, ***, $p<0.001$.

Figure S9

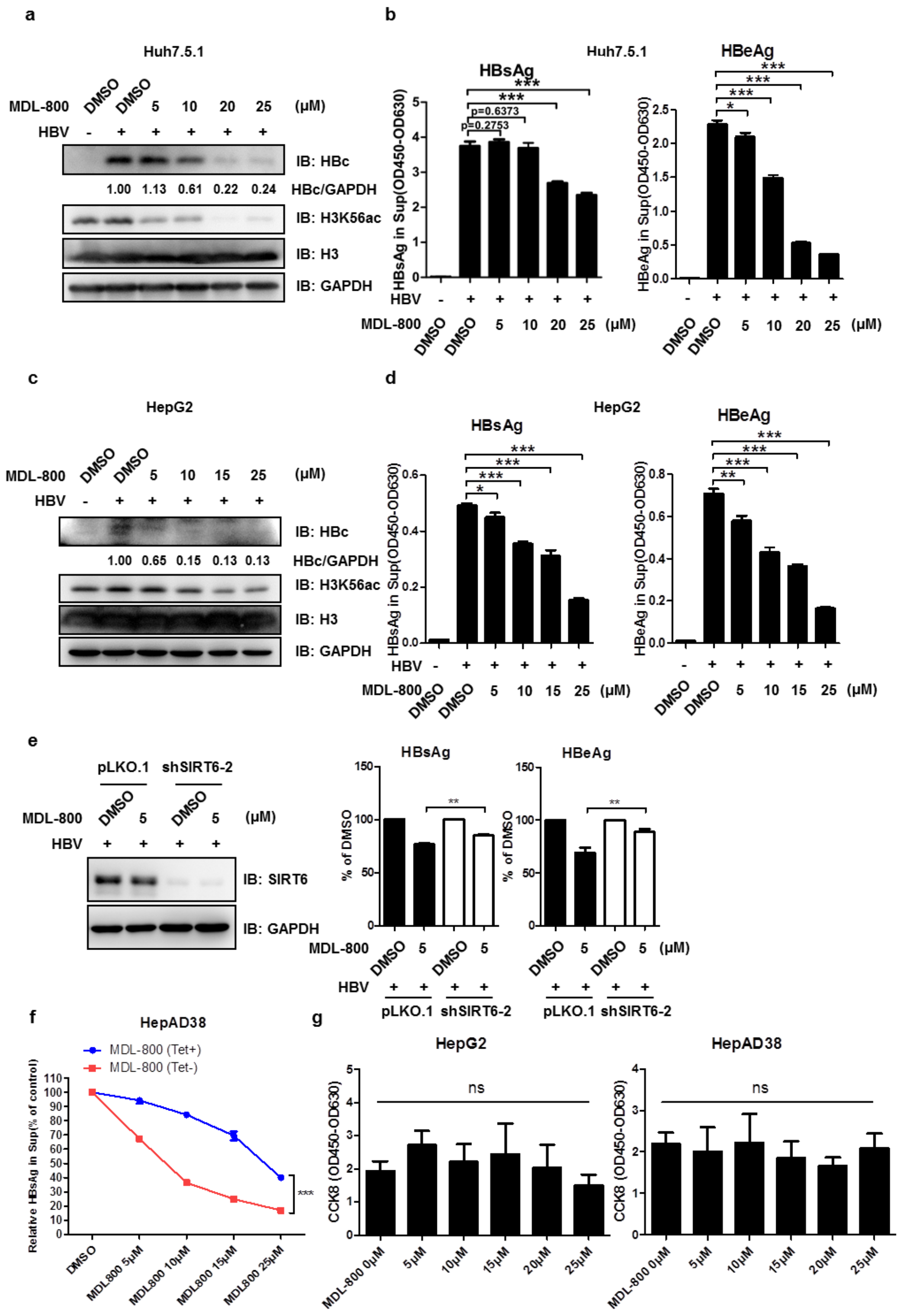

Figure S9: MDL-800 suppresses HBV gene expression in multiple cells. (a-d) Huh7.5.1 (a, b) or HepG2(c, d) cells were treated with increasing doses of MDL-800 after HBV transfection, the endogenous proteins were visualized with the indicated antibodies, while the levels of HBsAg or HBeAg in supernatants of cell cultures were determined with ELISA using anti-HBsAg or anti-HBeAg, n=3 for each group. **(e)**

The effectiveness of MDL800 on HBV was tested in SIRT6 knock-down HepG2 cell line or control HepG2 cell line. For ELISA, n=3. *, $p<0.05$, **, $p<0.01$, ***, $p<0.001$. (**f**) We performed the same experiment with tet present, and for comparison, the inhibition curve of MDL-800 without tet was also depicted in the same diagram, we had set the HBsAg level of DMSO control group as 100% and normalized each data point in the experiment to the DMSO group for the convenience of comparing the inhibition curve in different conditions. (**g**) As for MDL-800, we performed cell viability analysis using CCK8 in HepG2 and HepAD38 cells, the results shown that at lower range (<=25μM) cytotoxicity of MDL-800 is very low.

Figure S10

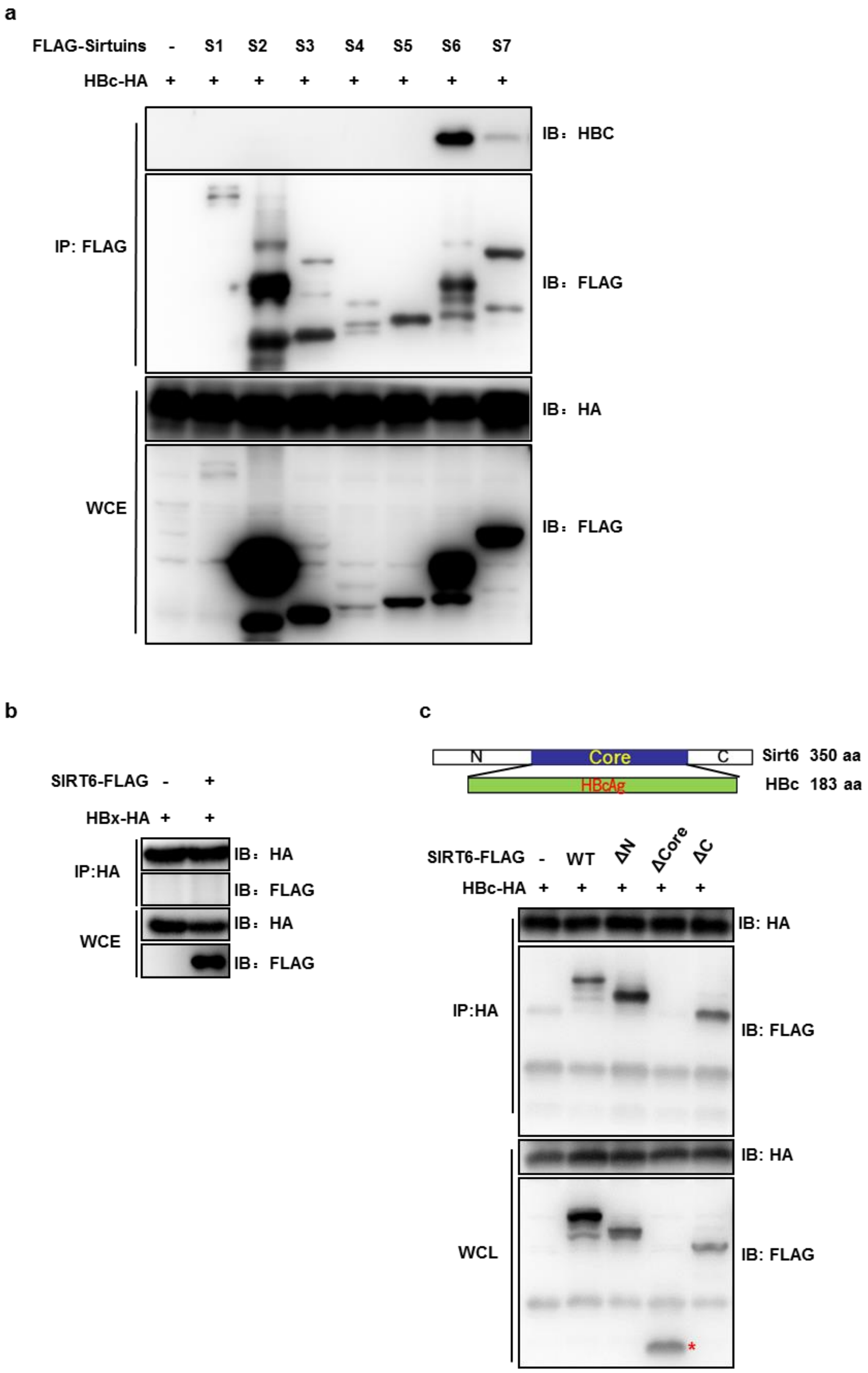

Figure S10: The interaction between SIRT6 and HBc. (**a**) HEK293T cells were transfected with HA-tagged HBc and FLAG-tagged Sirtuins family proteins, and co-immunoprecipitation was performed using anti-FLAG beads. (**b** and **c**) HEK293T cells were transfected with plasmids encoding HA-tagged HBx and FLAG-tagged

SIRT6, with cells harvested at 48 h.p.t (hours post transfection), and lysates were subjected to Co-immunoprecipitation (Co-IP) followed by IB with indicated antibodies. (**b**) SIRT6 did not interact with HBx. (**c**) 293T cells were co-transfected with plasmids encoding HA tagged HBc and FLAG-tagged full-length SIRT6 or the indicated fragments, after 48 hours, Co-immunoprecipitation (Co-IP) was performed. **ΔN,** SIRT6 deleted the N terminal domain (1-48Aa); **ΔC**, SIRT6 deleted the C terminal domain (272-328Aa), **ΔCore**, SIRT6 with the catalytic core domain (49-271 Aa) deleted. WCE: whole cell extracts. WCL: whole cell lysates.

Table S1. Patient information in figure 3i and 3j.

Patient Number	Clinical Diagnosis	Age	Gender	HBV DNA (copy/ml)	HBsAg (IU/ml)	Anti-HBs(mIU/ml)	HBeAg (S/CO)	Anti-HBe(S/CO)	Anti-HBc (S/CO)
1	Primary Liver Cancer Without HBV	61	Male	<50	0.01	0.01	0.35	0.08	0.5
2	Liver Cirrhosis Without HBV	58	Male	NA	0	108.94	0.28	0.34	7.38
3	Liver Cirrhosis Without HBV	48	Male	NA	0.03	0.69	0.26	0.03	9.23
4	Liver Cirrhosis Without HBV	71	Male	NA	0	3.54	0.33	1.42	7.92
5	Donor of Liver Transplantation	NA	NA	0	0	NA	0	NA	NA
6	Donor of Liver Transplantation	NA	NA	0	0	NA	0	NA	NA
7	Primary Liver Cancer With HBV	54	Male	NA	250	0.22	0.37	0.17	8.24
8	Primary Liver Cancer With HBV	66	Male	<50	99.78	0.01	0.273	0.04	9.74
9	Primary Liver Cancer With HBV	51	Male	<50	250	0.32	0.561	1.01	11.04
10	Primary Liver Cancer With HBV	53	Male	14500	250	0.06	0.294	0.01	11.79
11	Primary Liver Cancer With HBV	42	Male	8620	250	<0.01	0.899	0.92	9.92
12	Primary Liver Cancer With HBV	46	Male	5990	250	<0.01	0.335	0.01	12.02

Footnote: Patient 2-7 were diagnosed at Ruijin Hospital, others were diagnosed at Eastern Hepatobiliary Surgery Hospital.
NA, Not Available; IU, International Unit; S/CO, Sample Optical Density / Cut-off Value.

Table S1: Patient information in this study. Five most widely used HBV test for HBsAg, HBsAb, HBeAg, HBeAb and HBcAb were determined at indicated hospital. HBV DNA copy number in some patient serum was also measured. Note that patient 5 and 6 were donors of liver transplantation and tested for HBV free.

Data File S1. FPKM of ribosome profiling.

Data File S2. Ribocode analysis.

Data File S3. Novel peptides.

Data File S4. ncGRWD1 ncPON2 interactor.

Data File S5. FPKM of RNAseq.

Data File S6. DEGs list of RNAseq.

Data File S7. DEGs list of ribosome profiling.

Data File S8. Translation_efficiency.

Data File S9. PPP1R15A PGAM5 interactor.

Data File S10. proteome.

Data File S11. List of resources used in this study.
